# Examining the mental health outcomes of peer-led school-based interventions on young people aged between 4 and 18 years old: a systematic review protocol

**DOI:** 10.1186/s13643-019-1027-3

**Published:** 2019-04-26

**Authors:** Thomas King, Mina Fazel

**Affiliations:** Department of Psychiatry, University of Oxford, Warneford Hospital, Oxford, OX3 7JX UK

**Keywords:** Mental health, Well-being, Peer, Intervention, School, Child, Adolescent, Systematic review

## Abstract

**Background:**

Recent estimates suggest that one in ten young people worldwide experiences a diagnosable mental health disorder, with many more suffering subsyndromal levels of psychological distress. As young people spend much of their time in schools, the role of educational settings in the delivery of mental health provision is increasingly recognised. Advances in neurodevelopmental research have highlighted the important and complex role of peer influence on adolescent behaviours, suggesting peer-led support schemes have high potential utility. Schools worldwide have implemented peer-led interventions with mixed results, but the global evidence base on their effectiveness remains limited. This systematic review aims to examine the evidence base of the outcomes of school-based peer-led interventions on the mental health of young people aged 4–18.

**Methods:**

Eligible studies will be randomised controlled trials and observational studies that report on the mental health outcomes of a peer-led school-based intervention. Eligible participants will be aged 4–18 and will be current students of the intervention school. Individual- and group-based interventions will be included. The following 11 databases will be screened based upon their reach in healthcare and education: PsycINFO, MEDLINE, EMBASE, CINAHL, CENTRAL, BEI, Scopus, Web of Science, ERIC, SSCI and Social Care Online. There will be no restriction placed on publication period, original language or country of publication. Papers will be systematically screened for eligibility by two review authors. Data will be presented as a descriptive report. A meta-analysis will be carried out if a subset of studies allows, given the anticipated high levels of heterogeneity.

**Discussion:**

This systematic review will be the first to synthesise the global evidence on the mental health outcomes of peer-led interventions for children and adolescents in a school setting. It will analyse the available data in order to understand the role of these interventions in schools, inform future developers of peer support programmes and identify gaps in current research. This review will be of value to policy makers, health and education services, researchers and those involved in delivering peer support initiatives.

**Systematic review registration:**

PROSPERO CRD42018116243

**Electronic supplementary material:**

The online version of this article (10.1186/s13643-019-1027-3) contains supplementary material, which is available to authorized users.

## Background

It is estimated that over one in ten young people worldwide experiences a diagnosable mental health disorder [1, 2]. Many young people also experience issues with general well-being, requiring support without necessarily fulfilling diagnostic criteria [3]. Left untreated, mental health issues in adolescence can continue into adulthood and cause lasting and disabling effects [4]. Therefore, childhood and adolescence are seen as key periods of mental health intervention [3, 5, 6]. With this in mind, improving young people’s access to services is a main priority [3].

Specialist mental health services are in place for young people but are under increasing pressure, with evidence suggesting a rise in waiting times and high demand for limited resources [5, 7]. Young people may also face barriers to accessing these kinds of professional services, due to fear of stigma, feelings of embarrassment or poor symptom recognition [3, 8]. Many other young people find that they fall below the threshold for specialist care but still suffer the disruptive effects of their mental health experiences [9], with impacts including academic performance, peer and family relationships and self-esteem. This suggests the need for an integrated approach, whereby support systems are woven into other sectors, such as education, to address needs at different levels [3]. Given that young people spend much of their time in school, the development of this setting as a platform for mental health input is a key priority [3, 6].

An increasing number of schools are using peer-led interventions to better integrate mental health support into education [10]. These involve students providing support or education for other young people in their school. These interventions can address a range of issues, such as bullying, school transitions, physical health issues and others. The versatility of peer support makes it a difficult concept to define; however, widely used definitions focus on a number of key components: (1) the intervention is delivered by a peer, to a peer; (2) the intervention is delivered in a structured way, involving training and supervision for the supporters and involvement of the school; and (3) the intervention can be designed to provide education, support or information [11, 12]. Early studies have suggested mixed outcomes following these interventions, but the evidence base remains limited [10, 12, 13].

A proposed model of school-based mental health provision in high-income countries is a three-tiered approach to treatment. This includes (1) ‘universal’ interventions, which target all students in a school or classroom; (2) ‘selective’ interventions, targeting populations deemed at risk of developing mental health issues; and (3) ‘indicated’ interventions, which target young people already exhibiting mental health symptoms [6]. This model has an emerging and encouraging evidence base [14, 15]. Schools may elect to operate a peer support programme within the context of any one of these tiers; interestingly, a survey of peer support programmes in the UK suggested that secondary schools mostly operate targeted peer interventions, while primary schools favour a more universal approach [10]. A separate systematic review and meta-analysis of youth mentoring programmes across all school ages found particularly positive effects of interventions targeted at at-risk young people, though the included studies were not all ‘peer-to-peer’ or conducted within the school environment [16]. This opens up an interesting argument about the most suitable model for implementing peer support programmes in schools.

There are a number of factors that make peer-led interventions suitable to the school setting. Firstly, a major appeal is the potential for peer support programmes to benefit those giving and receiving the intervention, i.e. peer supporters and those they support, in areas such as social skills, communication and self-esteem [10, 13]. Secondly, schools implementing peer-led programmes can select from a large pool of potential peer supporters and, likewise, offer a very accessible service to a broad group of possible users. This could make access simpler and more readily available for young people and can reduce costs and resources for the school. These interventions also have the potential for scalability, that is, sizing up or down depending on demand.

Lastly, neurodevelopmental changes in the adolescent brain mean that peer relationships grow in importance and can strongly influence decision-making, suggesting social influences within their age group play a key role in behaviour [17, 18]. Therefore, young people may be more likely to access this kind of peer-led service compared to more traditional interventions with an adult professional [3]. Close peer support networks have been shown to buffer peer conflict and decrease risky behaviours [18], as well as help young people regulate their responses to stress factors [18] which, given the likelihood of stressors in the school context, justifies the consideration of more formalised school-based peer support systems. Conversely, however, young people have also been shown to display *more* risky behaviours when accompanied or observed by their peers [17], which suggests the adverse potential of some adolescent dynamics. The significant role of peer influence in adolescent decision-making is clearly complex and warrants further exploration in the context of delivering peer-led mental health interventions.

Examples of peer support programmes exist internationally in schools, colleges and universities. A survey of English primary and secondary schools conducted in 2009 [10], the most recent data available, suggested that around 60% of schools operate some kind of formal peer support scheme. Some of these programmes focus on mental health outcomes specifically, while others address issues more specific to the environment, such as academic attainment, school transitions and bullying. These programmes come under the banner of peer support, peer mentorship, peer counselling, peer buddying, peer befriending and more [10]. There is an identified need within the global literature to understand the connection between these common types and establish an overarching definition of the concept of peer-led intervention in the school context.

Despite the apparent popularity of these interventions, there has been no systematic review to date that specifically examines the mental health outcomes of peer-led interventions in schools.

However, similar reviews have been conducted which show mostly positive, yet inconclusive results. A meta-analysis by Whiston et al. [19] compared the efficacy of different school-based counselling interventions evaluated in both published and unpublished literature. Despite an overall significant average effect size, the authors could not provide clear evidence in support of peer facilitation and mentoring programmes as the outcome measures were largely heterogenous. The analysis may also be limited in its reach by only featuring studies published in the USA.

A separate systematic review and meta-analysis [16] looked at mentoring programmes in both school and community settings for ‘youth’ (pre-kindergarten to secondary school) using ‘non-parental adults or older youth’ as mentors. The study identified 73 eligible studies and a total of 83 separate intervention evaluations within them. Positive effects were observed for most domains, and benefits were particularly noted when ‘older youths’ were the chosen mentors, which supports the evidence for peer-led interventions. The review also identifies four conditions that can influence programme effectiveness: characteristics of youth, mentor recruitment and selection, criteria for matching youth with mentors and mentor-role expectations.

There is evidence of growing governmental interest in peer support. In 2014, the Department of Health published ‘Future in Mind’ [7], which highlighted the importance of mental health support in schools and focuses on peer support as a key area for development. In 2017, the Department for Education commissioned a review of the evidence for peer support programmes in schools [20]. The review found mixed evidence for efficacy but highlighted a lack of reliable data in the field and confusion over definitions and terminology. The report acknowledges the potential benefits of peer support interventions in schools. While not exhaustive, the review gives a helpful overview of some recent large-scale peer support projects and identifies gaps in the evidence base, such as long-term impacts, financial cost of interventions and sufficient comparison with other types of intervention.

### Objective of the current review

The evidence base around peer-led interventions in schools is emerging, and there is increasing national and international interest. It is therefore timely to better scrutinise the existing evidence base to understand the potential impact of this approach. This review aims to address this knowledge gap by (1) mapping the range of different peer-led, school-based interventions that have been evaluated in the literature and (2) critically examining the evidence base around the mental health and well-being outcomes of peer-led interventions in school-based settings. This protocol was developed using the PRISMA-P [21] checklist for guidance. The completed checklist can be found as an Additional file 1 to this document.

## Methods

### Participants

Studies will be included if they evaluate school-based interventions aimed at current pupils. Studies reporting outcomes for either peer supporter or peer service user (or both) will be eligible. Participants must be aged 4–18 years old (school-aged) and access the intervention in a primary, secondary, special education or college setting. Participants with or without pre-existing psychological, emotional and behavioural difficulties will be included. Studies reporting outcomes on a minimum of 20 participant pairs will be included.

### Study design

Any randomised controlled trials (RCTs) or observational studies, e.g. case-control and cohort studies, that evaluate the mental health outcomes of a peer-led school-based intervention will be included. Eligible studies do not need to have a control group as long as a pre- and post-test evaluation is included.

### Setting

Eligible studies must include a peer-led intervention for school-aged children administered in a school setting. Individual- and group-based interventions will be included. Interventions delivered online will be included if there is a peer supervisor/trainer involved.

### Primary outcomes

This review will look at the mental health and well-being impact of peer-led school-based interventions for young people where evaluations have followed structured measurement criteria. A variety of tools are available to assess diagnosable mental disorders, such as depression and anxiety, and more general well-being states, which include headings such as self-esteem, security and confidence. These assessments can be completed either by a trained practitioner or through self-reporting. This review will assess mental health and well-being outcomes based upon the results of the specific tools used in each study.

### Intervention

#### Inclusion criteria

Eligible studies will have conducted an evaluation to measure the mental health impact of a peer-led intervention for young people in a school setting. ‘Mental health’ here incorporates both psychological well-being and pathological disorders; as assessment tools for both of these conditions vary widely, any evaluation that reports on mental health changes in either context will be included in this review. Eligible studies must include an intervention that took place within the school setting. One-to-one and group-based interventions will be included, as long as the group intervention is primarily led by a school-aged peer. Studies evaluating interventions aimed at broader issues that may also affect mental health, such as bullying, LGBTQ+ and school transitions, will be included if they report on mental health outcomes. Evaluations of peer mentor training programmes where impact on participants’ mental health is measured will be included, as well as educational peer-led interventions where mental health outcomes are reported. Both quantitative and qualitative studies will be included.

#### Exclusion criteria

Quantitative studies that do not use an evaluation tool to measure mental health outcomes will be excluded. Interventions for young people led exclusively by a professional facilitator, such as in certain examples of group work, will also be excluded.

### Comparator/control

Peer-led intervention versus no intervention, non-peer-led intervention or different types of peer intervention

### Search strategy

Sources will be derived through systematic searches of the following 11 electronic databases: PsycINFO, MEDLINE, EMBASE, CINAHL, CENTRAL, BEI, Scopus, Web of Science, ERIC, SSCI and Social Care Online (Additional file 2). These databases have been chosen based on their capture of mental health and education outputs. Relevant conference websites, Web of Science conference proceedings, clinicaltrials.gov and the online database PsycEXTRA will be searched separately to locate online grey literature.

Key search terms extracted from the research question will include mental health, peer, school and intervention. From these, an extensive list of synonyms and related terms will be developed using existing studies on this subject and database thesaurus tools, as well as consultation with experts in the field, including research librarians. Broad search terms such as ‘mental health’ will be ‘exploded’ within the database to include all variations that are relevant to the review. An example of the search strategy can be found as a supporting document.

Hand searches will also be carried out for eligible studies using reference lists of included articles and related systematic reviews, as well as those articles that have cited eligible studies online. Principal authors of relevant studies will be contacted where further detail of their study is sought. A PRISMA flow diagram [22] will be maintained throughout the literature search process in order to track total records at each stage.

There will be no restriction placed on the publication period, original language or country of publication. Any protocol amendments will be justified and documented in a protocol addendum and in the final report.

### Data extraction

EndNote software will be used to manage studies and remove any duplicates. In order to reduce the risk of selection bias, both reviewers will screen the titles and abstracts of studies identified following the initial search as outlined above. Once these have been filtered, each reviewer will then independently read the full texts of all remaining studies in order to assess them for relevance. Any ineligible studies will be discarded. Any disagreements between the two reviewers will attempt to be resolved through discussion. Where no consensus is reached, a third reviewer will be consulted.

Extracted information include the following: author, title and year of study; country; school characteristics; peer characteristics; mentor characteristics; study aim; study design; study methodology; type of intervention; target outcomes; reported outcomes; measurement/evaluation tool; population age; population gender; sample size; presence or absence of control group; control conditions and risk of bias.

### Strategy for data synthesis

The reviewer will present a descriptive report of the findings according to the types of interventions identified and the efficacy of those interventions.

Initial exploration indicates that there will be high heterogeneity in the studies; however, if an appropriate subsample can be identified, then we will conduct a meta-analysis on this subsample. This will, for example, be if the same peer-led programme is analysed using the same or similar measures in different samples.

### Risk of bias and quality assessment

The risk of bias of eligible studies will be assessed using the Cochrane Risk of Bias Tool [23] for RCTs and the Newcastle-Ottawa Scale [24] for observational studies. These have been chosen due to their applicability to the two chosen methodologies for review and wide usage and regard. Each reviewer will perform an independent and blind risk of bias assessment for each included study.

### Confidence in cumulative evidence

The strength of the body of evidence will be assessed using the Grades of Recommendation, Assessment, Development and Evaluation (GRADE) system [25, 26].

### Dissemination

The findings of this review will be submitted for publication in a peer-reviewed journal.

## Discussion

This systematic review will be the first to synthesise the global evidence on the mental health outcomes of peer-led interventions for children and adolescents in a school setting. It will analyse the available data in order to inform future developers of peer support programmes, to identify gaps in current research and to move towards a more nuanced understanding of the role of peer-led interventions in schools. Two key issues of suitability and scalability will be addressed. This review aims to provide a knowledge base that will have practical application in the improvement of mental health support for children and adolescents in schools. This review may be of value to policy makers, researchers and those involved in delivering peer support initiatives.

## Additional files

Additional file 1: PRISMA-P 2015 checklist. (DOCX 33 kb)

Additional file 2: Example search—PsycINFO. (DOCX 15 kb)

## Abbreviations

GRADE: Grades of Recommendation, Assessment, Development and Evaluation
LGBTQ+: Lesbian, Gay, Bisexual, Transgender, Questioning +
NIHR: National Institute for Health Research
PRISMA: Preferred Reporting Items for Systematic Reviews and Meta-Analysis
PRISMA-P: Preferred Reporting Items for Systematic Reviews and Meta-Analysis Protocols
RCT: Randomised controlled trial
